# Olfaction, Vision, and Semantics for Mobile Robots. Results of the IRO Project

**DOI:** 10.3390/s19163488

**Published:** 2019-08-09

**Authors:** Javier Monroy, Jose-Raul Ruiz-Sarmiento, Francisco-Angel Moreno, Cipriano Galindo, Javier Gonzalez-Jimenez

**Affiliations:** Machine Perception and Intelligent Robotics group (MAPIR), Dept. of System Engineering and Automation Biomedical Research Institute of Malaga (IBIMA), University of Malaga, 29071 Málaga, Spain

**Keywords:** robotics, robotics olfaction, chemical sensors, gas source localization, e-nose, gas distribution mapping, object recognition, semantic networks, machine learning, ontology

## Abstract

Olfaction is a valuable source of information about the environment that has not been sufficiently exploited in mobile robotics yet. Certainly, odor information can contribute to other sensing modalities, e.g., vision, to accomplish high-level robot activities, such as task planning or execution in human environments. This paper organizes and puts together the developments and experiences on combining olfaction and vision into robotics applications, as the result of our five-years long project *IRO: Improvement of the sensory and autonomous capability of Robots through Olfaction*. Particularly, it investigates mechanisms to exploit odor information (usually coming in the form of the type of volatile and its concentration) in problems such as object recognition and scene–activity understanding. A distinctive aspect of this research is the special attention paid to the role of semantics within the robot perception and decision-making processes. The obtained results have improved the robot capabilities in terms of efficiency, autonomy, and usefulness, as reported in our publications.

## 1. Introduction

The sense of smell is not the most vital one for humans, but we certainly use it every day. When we face a cup with a dark-colored liquid, we can assure that it is a cup of coffee not only from what we observe, but also from what we smell. When we detect an alarming odor that might be associated to gas/butane, we do not look for the possible escape in the living room but we firstly go to the kitchen, where we do not inspect randomly, but we turn our attention to those devices that use gas (e.g., hob, oven, etc.). As in the last example, the smell sense usually triggers alerts: a possible fire, a gas leak, food in poor condition, etc., but it is also associated to emotionally rooted processes [1]: memories, attraction or repulsion, etc. Both facets are interesting in robotics, although the latter, especially relevant in the long term for the so-called *social robots* [2,3], is beyond the scope of our current research. The IRO project focuses on the usefulness of a mobile robot able to detect and measure gases in the environment in order to identify the activities carried out in its surroundings, e.g., smoking, cooking, mopping the floor, etc. Having identified the situation, the robot should be able to act consistently, for example, locating and scolding the smoker, avoiding to pass by freshly mopped areas or, perhaps, interacting in a social way to help the person who is cooking. Some related works in this field [4,5] present mobile robots endowed with olfactory capabilities and applications to detect odor sources. The work done within the IRO project combines olfaction with vision and semantic knowledge to improve the robot abilities, which differs from such related works. To provide a mobile robot with olfaction capabilities, we relied on electronic noses (e-noses) [6], i.e., electronic devices composed by a set of gas sensors and different software components that provide a measure of the type and concentration level of the detected volatile substances. Despite the important advances in recent years in the development of this technology, the performance of gas sensors and algorithms for the classification of gases is still far from the olfactory capacity of humans, not to mention some other animals with much more developed olfactory capabilities. Despite this limited performance, the olfactory information interestingly increases the robot abilities when combined with other sensors such as vision, and knowledge sources such as semantics. For example, if the robot detects smoke, the utilization of vision would be crucial for identifying an oven and inspecting it as the possible object releasing the alarming gas. Additionally, semantic information regarding the usual location of ovens, i.e., kitchens, can improve the robot actuation.

The ultimate goal of our research is twofold: to enable a mobile robot to combine olfaction and vision information, and to exploit semantic knowledge to smartly operate within human environments. Although the individual results of the project have already been published elsewhere [7,8,9,10,11], this paper contributes an overall and comprehensive view of all its findings and results by: (i) summarizing the performed experiments; (ii) describing the different setups for both the gas recognition and classification tasks, as well as for the combination of artificial vision and olfaction to generate semantic information; and (iii) finally analyzing the potential advantages that e-noses can provide for gas detection and scene understanding. Therefore, this document comprises in one place all the knowledge built from the IRO project.

## 2. Project Overview

The general objective of the IRO project is to investigate mechanisms for integrating olfactory data into the robot sensing system, as well as the development of algorithms for decision making and task generation that exploit the combination of the different sensor modalities. The key idea behind our research here is that the perception of gases, including both their classification and the measurement of their intensity or concentration, can improve the intelligent behavior of the mobile robot, upgrading its performance in terms of efficiency, autonomy and usefulness. Within this global target we can distinguish three partial objectives:

- **Design and fabrication of an artificial nose (e-nose) adapted to the requirements of a mobile robot.** Most of the e-noses used in mobile robotics are designed for measuring only the chemical concentration, aiming at tasks such as the creation of concentration maps and/or the search of the emission sources. In the context of the present project, it is necessary that the electronic nose is designed to also provide information on the type of gas, that is, be as effective as possible in the classification of the detected chemical volatile. The objective is, therefore, to combine both facets which requires integrating different sensor technologies into a single device.
- **Gas classification and object recognition for robotics applications.** The robot, equipped with a vision system (e.g., one or multiple RGB or RGB-D cameras) and an electronic nose, could successfully improve the vision-based recognition of simple objects, exploiting the odor information gathered in the surroundings, as well as enhancing the gas classification when considering the semantic information and the probabilistic categorization of the detected object.
- **Exploiting high-level olfactory and visual semantic information in the planning and execution of tasks.** Semantics provide additional human-like information to the perceived elements. For example, a high concentration of gases related to rotten food suggest that somebody forgot about it. Semantic information can be exploited to automatically infer new robot tasks in order to maintain a set of pre-stablished human-like norms, in this case, rotten food should be taken out of the house [12]. Among the multiple tasks that can benefit from such inference process, we focus on the challenging task of source localization with a mobile robot in indoor environments, aiming at minimizing the necessary time to locate the object emanating the gases in the environment.

The following sections describe with more detail the work done to reach these partial objectives. Section 3 describes the hardware involved in the project, both the electronic noses and the employed mobile robots. Then, Section 4 summarizes the classification algorithms considered to recognize different gases, analyzing the impact of the robot movements in the gas recognition. Finally, Section 5 and Section 6 present our insights on combining olfaction, vision, and semantics abilities in mobile robotics.

## 3. Hardware Description

This section describes the hardware components employed in the set of experiments performed during the IRO project, with a particular emphasis in the e-noses and the mobile platforms used to carry them.

### 3.1. Electronic Noses

E-noses are devices designed to detect, measure and classify volatile chemical substances by means of an array of gas sensors. Commonly, the gas sensors employed react to a wide range of different gases (non-selective), but provide no specific information about the chemical identity. Therefore, the output of the sensor array is usually further processed by some sort of machine learning algorithm to classify [10,13] or quantify [14,15] the samples. However, it must be noticed that in the last decade multiple advances have been made towards developing selective gas sensors [16,17], which could reduce the complexity of e-noses in a close future by reducing the number of sensors to host and the need of a post-processing stage to classify the gases. As a result, e-noses offer a relatively cheap and fast tool to assess the presence of gases, but with a substantially greater error and uncertainty margin than precise analytic methods, such as gas-chromatography or mass-spectrometry [18].

Common gas sensor technologies employed to build e-noses include Metal OXide (MOX), Amperometric ElectroChemical (AEC), Quartz Crystal Microbalance (QCM), Conducting Polymers (CP), and Surface Acoustic Wave (SAW). Each of these exhibits advantages and disadvantages in terms of selectivity, sensitivity, response speed, influence by environmental conditions and drift over time, among others [6,19]. However, no single technology excels in all categories. Thus, limiting the design of an e-nose to a single sensor technology will restrict its performance and, quite often, prevent it from reaching the demanded specifications [9]. This motivates the combination of different gas sensor technologies into a single e-nose, which would result in a sensor array with better dynamic capabilities and a more informative output than any single sensor technology. Since it is unfeasible to install all possible gas sensors and technologies simultaneously on a single device, it also becomes appealing to design an e-nose in such a way that its sensor array can be reconfigured depending on the applications, keeping it cost-efficient and compact.

To attain the objectives identified in this project, our first step has been the design and fabrication of e-nose prototypes for gas classification and concentration estimation, as well as their posterior integration into a mobile robot. In the earliest stages of the project, we employed the so-called Multi-Chamber Electronic (MCE) nose, developed in one of our previous works [20]. The MCE nose is a device that comprises several identical sets of MOX sensors accommodated in separate chambers so that it can alternate between sensing and recovery states, providing, as a whole, a device capable of sensing changes in chemical concentrations faster than conventional e-noses. This overcomes the main drawback of MOX sensors in terms of recovery time after being exposed to gases, which highly restricts its usage in applications where the gas concentrations may change rapidly, as in mobile robotic olfaction.

**Figure 1:**
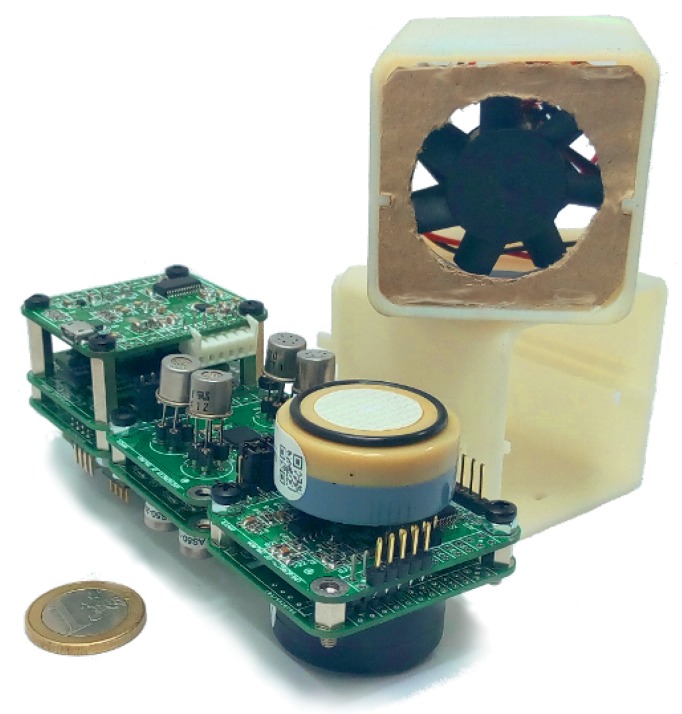
Picture of the e-nose prototype built for the IRO project. Its modular and compact design allows it to be easily mounted on a mobile robot and adapted to the application requirements.

In subsequent stages, we exploited our experience with the MCE nose and proposed, as a central contribution for the IRO project, a novel e-nose architecture [8] that combines self-contained and intelligent sensor boards (i.e., modules) with a decentralized design offering a viable solution to the problem of integrating heterogeneous gas sensors in a modular fashion. This allows us to create different and specific gas-sensing devices from inter-connectable building blocks, which not only brings versatility and reusability to the design of e-noses but also reduces development costs and ensures long-term serviceability, as new sensors can be added as needed. Moreover, the proposed e-nose architecture also enables the integration of other electronic components such as GPS for geo-referenced measurements, or wireless communications for remote readings, a feature which, despite not being a technological contribution, provides an improvement over most commercial e-noses and facilitates applications of mobile robot olfaction. Figure 1 shows a picture of the prototype built along the course of this project. The particular configuration shown includes a power module (along with a 2200 mAh lithium battery, useful for pre-heating the gas sensors when the robot is still not powering the e-nose), an SD memory card module to keep a log of all measurements, and four gas-sensing modules (hosting eight MOX sensors and two electrolytic sensors).

In terms of consumption, due to its modular nature, the total power needed by this e-nose is highly dependent on its particular configuration. As an example, the setup shown in Figure 1 has a maximum power consumption of ∼2.5 W, which is suitable for being supplied through a standard USB2.0 port. This value is low enough to not significantly compromise our robot’s autonomy, as they include high capacity lithium-ion batteries capable of powering all the electronic and mechanical devices on the robot, including the on-board PC and the wheels’ motors. A thorough description of the power consumption values for each module can be found in [8].

### 3.2. Mobile Robots

Along the course of the IRO project two different robotic platforms have been employed for carrying out the multiple experiments, namely *Rhodon* and *Giraff*.

**Figure 2:**
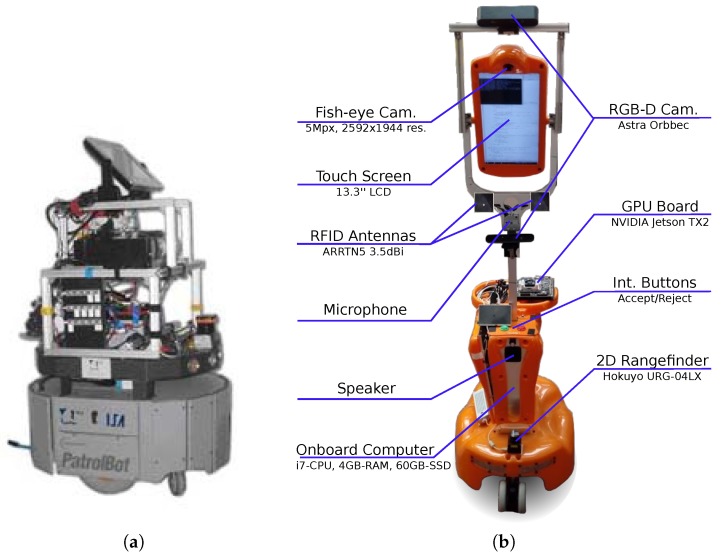
The robots employed in the experiments: (**a**) Rhodon; and (**b**) Giraff.

- *Rhodon* is a laboratory robot built upon a commercial PatrolBot platform (refer to Figure 2a), capable of being tele-operated or even to autonomously navigate (i.e., self localization and obstacle avoidance) by using a pair of 2D laser scanners: a SICK PLS (front) and a Hokuyo URG (back). The on-board PC controls both the navigation and data acquisition by means of a set of software modules running within a ROS framework. Since the experiments described in this paper corresponds to different stages of the IRO project and aimed to different purposes, diverse robot setups have been adopted, as specified in Section 4. The *Rhodon* robot has been available from the beginning of the IRO project, and is capable of carrying heavy loads, becoming ideal for the attachment of a robotic arm used in one of the experiments.
- The second robotic platform employed is the so called *Giraff* robot [21,22]. It has been used during the experiments regarding object recognition, as described in Section 6. In a nutshell, it is a telepresence robotic platform equipped with a frontal 2D laser range finder for navigation and localization, and a set of RGB-D cameras to capture 3D information from the environment (see Figure 2b). The *Giraff* robot became available later during the project and, as it is lighter and easier to transport than *Rhodon*, it was chosen for the experiments related to semantics, due to the need for recording visual measurements in a real house.

## 4. Gas Recognition and Classification for Robotic Applications

The task of odor recognition deals with the problem of identifying a volatile sample among a set of possible categories [23]. This process plays an important role in the development of many applications, such as city odor mapping [24,25], pollution monitoring [26], breath analysis in clinical environments [27], or the nowadays common estimation of blood alcohol content for drivers [28,29]. Among them, there are some applications such as pollution monitoring or leak detection that require measuring the environment continuously and/or at different locations. For such scenarios, the use of a mobile robot with the capability of identifying and measuring the volatiles’ concentration is of great help, as already reported in [30].

### 4.1. Gas Classification

The classification of volatile substances is, possibly, the most studied application of e-noses. Traditionally, this has been performed by analyzing the response of an array of gas sensors when exposed to pulse-like gas excitation under well-controlled measurement conditions (i.e., temperature, humidity, exposure time, etc.). Unsurprisingly, dozens of works report less than 10% classification error rate under these specific circumstances. However, when the classification is to be performed on a real, uncontrolled scenario, and particularly for the case where the e-nose is collecting samples on board a moving platform, assumptions such as a perfect alignment or equal length of patterns do not hold [31]. This, which is due to the dynamic and chaotic nature of gas dispersal, together with the strong dynamics shown by most gas sensor technologies, notably increases the complexity of the classification problem [7].

### 4.2. Continuous Chemical Classification

The discrimination of gases performed with a robot equipped with an array of gas sensors presents a number of additional challenges when compared to standard identification applications. While standard classification tasks usually host gas sensors inside a chamber with controlled humidity, temperature and airflow conditions, in robotics olfaction, there is no control over the sensing conditions. This entails that the sensor signals to be processed are noisy and dominated by the signal transient behavior [32]. Under these challenging conditions, chemical recognition can be seen as a particular case of time series classification, characterized by working on sub-sequences of the main data stream (see [33] for a complete review). Nevertheless, most of these approaches are proposed for uni-variate time series, while e-nose data are fundamentally multi-variate (i.e., based on an array of gas sensors with different dynamic responses). This, together with the aforementioned challenges of real data, make most segmentation approaches difficult to apply to e-nose data, which, in turn, affect negatively the classification rate.

**Figure 3:**
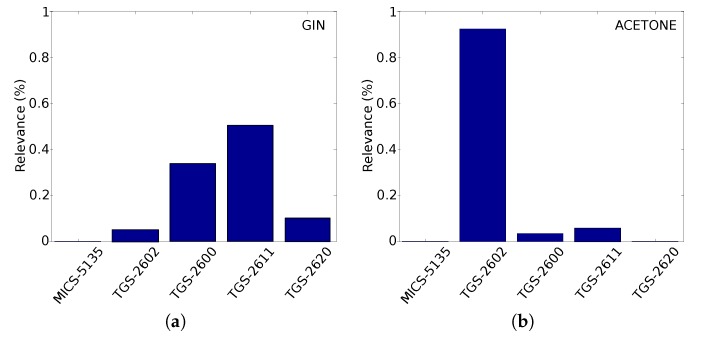

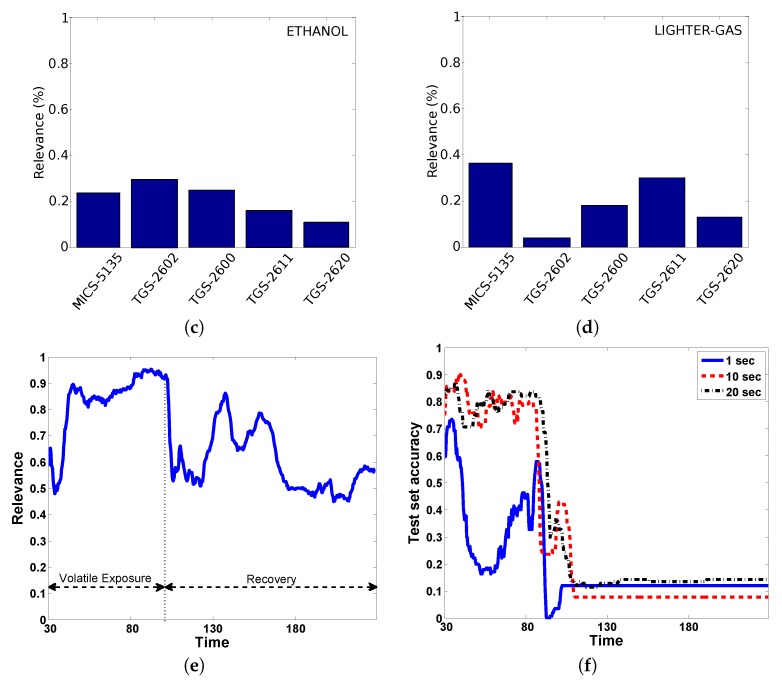
Illustration of the sensor and time points relevance for the classification of gases: (**a**–**d**) normalized sensor relevance of an e-nose composed of five gas sensors when exposed to four different gas classes; (**e**) time points relevance profile averaged over all classes; and (**f**) mean prediction accuracy over time for window lengths of ≈{1,10 and 20} s. These results correspond to an e-nose dataset collected under semi-controlled measurement conditions as described in [34].

**Figure 4:**
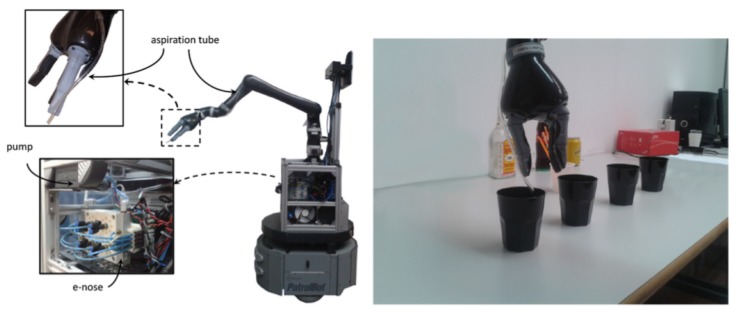
The Rhodon robot with the robotic arm used in the experiments.

A novel approach was published in [34] as a partial result of the IRO project to address the aforementioned issues. This approach is based on generative topographic mapping through time (GTM-TT) and integrates supervised classification and relevance learning (SGTM-TT) to the problem of volatile identification in mobile robotics. By exploiting the strong temporal correlation of the e-nose data, the method is capable of classifying gases with high accuracy employing short data sequences (1 s, 10 s and 20 s). Given the ephemeral nature of gas dispersion, the impact of the data sequence length on the classification performance is also analyzed, trying to push the limits towards a fast-response chemical recognition system. Furthermore, another remarkable advantage for robotics applications is the introduction of a relevance value, by studying the relevance of the different sensors composing the e-nose and the time points in the data sequence for predicting the class label. Figure 3 shows an example of these magnitudes for an e-nose composed of five gas sensors (Figaro TGS-2600, TGS-2602, TGS-2611, TGS-2620, and MiCS-5135) when exposed to four different gaseous substances (gin, acetone, ethanol and lighter-gas). As can be seen, the relevance in the classification process of each sensor drastically varies according to the gas being exposed, sometimes being one sensor dominant over the others, while in other cases it would be necessary to consider a combination of their outputs to achieve a good classification rate. Related to the time points relevance (Figure 3e), it can be seen how the most relevant data match the exposure time, while the relevance decays considerably during the recovery phase. However, due to the different recovery times of the sensors composing the e-nose, we can find some time-periods with high relevance that could also be used to get a high accuracy in the classification. In these experiments, the Rhodon robot was equipped with a robotic arm that held an aspiration tube connected to the MCE nose, as can be seen in Figure 4.

**Figure 5:**
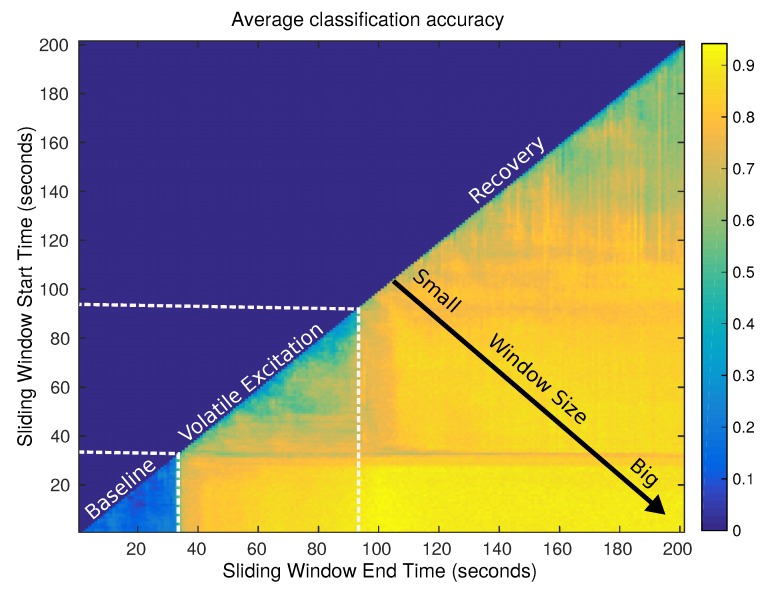
Average classification accuracy of a naive Bayes classifier for different lengths and positions of the sliding window within the time-series e-nose data.

Later, in [7], we advocated the use of the well known sliding window approach to avoid feature based segmentation and to study up to which extent considering delayed samples contributes to exploit the temporal correlation of e-nose’s data. This technique is attractive because it is simple, intuitive, and, moreover, amenable to online applications, which is a primary focus of the IRO project. We analyzed the impact of the window length on the classification accuracy (see Figure 5) for three state of the art classifiers, a variety of experimental scenarios, e-nose configurations and gas classes (employing three different olfaction datasets). The main conclusion of such work is that, for online chemical classification in uncontrolled environments, feeding the classifiers with additional delayed samples leads to a small, yet important, improvement (up to 6% units) on the classification accuracy.

### 4.3. Gas Classification in Motion

Having demonstrated that online chemical classification is feasible with a mobile robot, IRO also investigated the impact of carrying such task while the robot is navigating. We analyzed the induced changes in the gas sensor’s response and determined that the movement of the robot has an important impact on the classification accuracy if not properly considered, resulting in a decrease of up to 30% in some configurations [35]. We supported our conclusions with an extensive experimental evaluation consisting of a mobile robot inspecting a long indoor corridor with two chemical volatile sources (ethanol and acetone) more than 240 times, at four different motion speeds: low ≈ 0.2 m/s, medium ≈ 0.4 m/s, high ≈ 0.5 m/s and very high ≈ 0.6 m/s. In these experiments, apart from the e-nose, the Rhodon robot was equipped with a Gill WindSonic ultrasonic anemometer for measuring the wind flows in the environment, and a miniRAE Lite photo ionization detector as an alternative gas detector. The on-board e-nose, in turn, was composed of an array of 10 MOX gas sensors including Figaro TGS26xx sensors for measuring gases such as hydrogen, ethanol, CO or Iso-butane, and Hanwei MQx sensors for other substances such as LGP, propane or natural gas. This e-nose provided gas readings at a rate of 5Hz. Further information about the dynamic conditions of these experiments can be found in [35].

**Figure 6:**
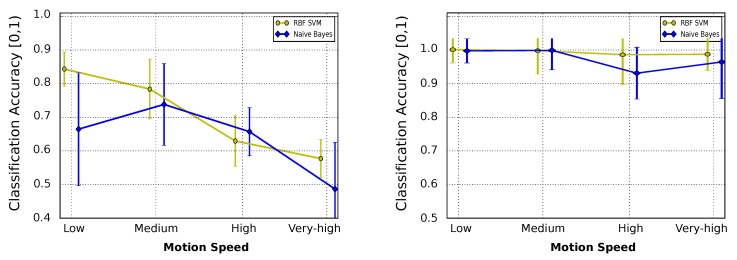
Average classification accuracy for different motion speeds using two classifiers (Naive Bayes and RBF SVM (Radial Basis Function for Supported Vector Machine)): (**Left**) classification accuracy when training the classifiers with static data samples; and (**Right**) results when the classifiers have been trained with data collected in motion.

To analyze to which extent the motion of the gas sensing device may affect the classification accuracy, we trained multiple classifiers with samples of each chemical volatile collected in a traditional static setup (i.e., both robot and gas source standing still), and then, analyzed the classification performance for a set of increasing motion velocities. Figure 6 (left) shows the results of the experiments from which a noticeable reduction in the classification accuracy is observed when increasing the motion speed. This confirms our suspicions about the negative impact that the motion speed of the robot has over classification rate.

To overcome, to a certain degree, the aforementioned effect, we also analyzed the classification accuracy when the classifier is also trained with in-motion data samples, proposing different training schemes. We showed that training a classifier with data collected in motion yields, on average, more accurate outcomes (see Figure 6, right) than using a static setup (Figure 6, left). Moreover, we found that it is not necessary to train the classifiers with data gathered at the same speed than the testing data to remove this negative correlation, but it suffices to capture the underlying dynamics. As a general conclusion, the absolute speed is not a determinant parameter, but the gap between the speeds used to collect the training and testing datasets is an aspect to be taken into consideration when deploying real olfaction applications with a mobile robot.

## 5. Object Recognition and Semantic Knowledge for Robotic Applications

From the object recognition side, the peculiarities of the acquisition process of visual data by a mobile robot permits the inspection of larger portions of the robot workspace, gathering rich semantic information. In this case, semantic information comes in the form of contextual relations, i.e., objects that are found according to certain configurations: keyboards are usually in front of computer screens, microwaves are in the same room as refrigerators, tables are typically surrounded by chairs, etc. [36]. Thereby, during the object recognition process, the presence of a refrigerator in a room helps to disambiguate the classification of a white, box-shaped object as a microwave and not as a night stand [11,37].

**Figure 7:**
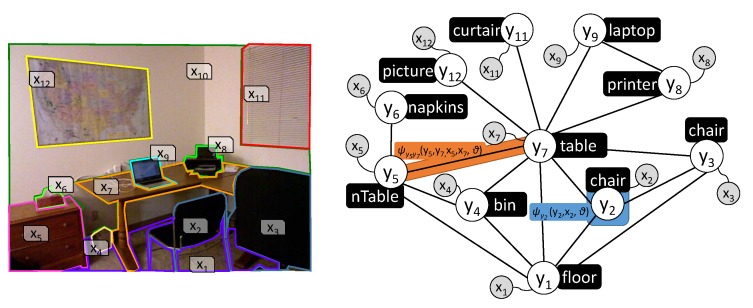
(**Left**) Scene from the NYUv2 dataset with segmented patches and their *ids* ($x_{1}\cdots x_{12}$). (**Right**) Conditional Random Field (CRF) graph built according to the patches in the NYUv2 scene (the node and relations of the wall, $x_{10}$, have been omitted for clarity). The orange area illustrates the scope of a pairwise factor modeling the relations between two objects, while the blue one stands for the scope of a unary factor classifying an object according to its features. Black boxes represent the expected results from a probabilistic inference process over such CRF.

**Figure 8:**
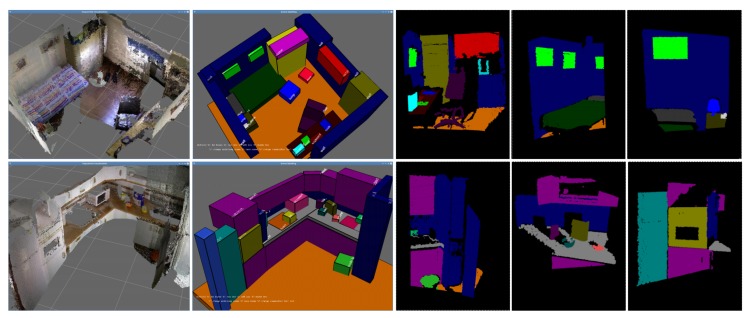
Examples of information from the Robot@Home dataset. The first column presents reconstructed scenes from the sequences within the dataset. The second column shows labeled reconstructed scenes. The third to fifth columns are examples of individual point clouds from RGB-D observations labeled by the propagation of the annotations within the reconstructed scenes.

To exploit these contextual relations in the IRO project, we make use of Conditional Random Fields (CRFs), a model from the Probabilistic Graphical Models (PGMs) family [38], and combine them with ontologies [39] to achieve a more robust performance. CRFs represent the objects in the environment as nodes in a graph, where edges are used to link contextually related objects (Figure 7). In [40], a survey on different learning approaches for these models is presented, performing a comparative analysis focusing on the time needed for training and the achieved recognition accuracy. This analysis is especially targeted at finding the most suitable one for scene object recognition, providing Loopy Belief Propagation (LBP) the best results [41]. These comparisons were done with two state-of-the-art datasets, including a particular one, called Robot@Home [42], specifically conceived to serve as a testbed for the evaluation of semantic mapping algorithms, mainly those exploiting contextual information (see Figure 8).

To combine different sources of contextual information, novel environment representations can be used such as the so-called Multiversal Semantic Map [43]. This map is an extension of traditional semantic maps for robotics [44], with the ability to coherently manage uncertain information coming from, for example, object recognition or gas classification processes, and reference them to the location where they were acquired into a metric map. Additionally, it also comprises semantic information codified by means of an ontology, enabling the execution of high-level reasoning tasks [45], which are of special interest in this project.

## 6. Exploiting High-Level Olfactory and Visual Semantic Information in the Planning and Execution of Tasks

Mobile robots operating in human environments such as offices, hospitals, or factories benefit from the fusion of different sensing modalities to efficiently accomplish tasks that are hard or even unfeasible to address if only one sensor is employed [46]. As mentioned, in the IRO project we focus on two of these modalities, namely vision and artificial olfaction, and study their application to a challenging problem: the localization of gas emission sources within real-world indoor environments, commonly referred as gas source localization (GSL) [47]. For that, the robot would need not only to detect the volatile chemical substance that is being release, but also pinpoint the location of its release source. As stated, enriching the search process with visual sensory information and considering semantic relationships through an inference process will enhance the current state of art of GSL algorithms.

To demonstrate this claim, two parallel approaches were considered: on the one hand, we relied on human intervention by means of a teleoperated mobile platform [48], delegating the inference of the most likely source location to the human tele-operator, and, on the other hand, we developed a fully autonomous system able to infer the most likely source location based on the sensory data available on the robot and high-level semantic reasoning [49]. Both approaches are detailed in the following sections and were assessed through experiments with the Giraff mobile robot.

### 6.1. Olfactory Telerobotics

**Figure 9:**
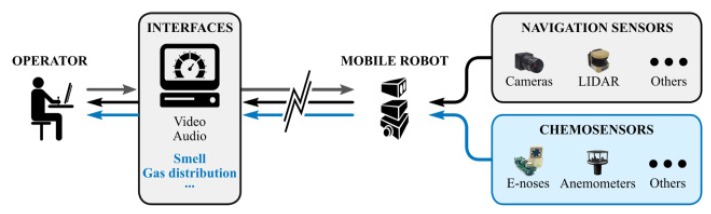
Diagram of a traditional teleoperation system (in black) and extended olfactory telerobotics (in blue). The latter requires equipping the mobile robot with additional sensors (e.g., an e-nose or an anemometer), and enhances the teleoperation user-interface to display this new sensory data.

Since inferring the type of object (and the location in the environment) of the gas source that is releasing the gases that have been detected by the robot is not straightforward, we simplified the problem by introducing the human factor and its powerful reasoning capabilities to solve this challenging problem [50]. In this context, *olfactory telerobotics* can be seen as the augmentation of the sensing capabilities of a conventional teleoperated mobile robot to acquire information about the surrounding air (i.e., gases, wind-speed, etc.) in addition to the usual audio and video streams (see Figure 9).

**Figure 10:**
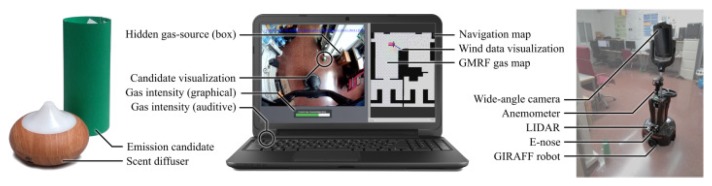
(**Left**) Ultrasonic scent-diffuser and one of the gas source candidates. (**Middle**) User interface for teleoperating the robot running on a laptop. (**Right**) Giraff telepresence-robot equipped with an e-nose and an anemometer for remote sensing, and a LIDAR for self-localization.

To evaluate whether the human reasoning can be exploited through a teleoperated robot to efficiently locate the gas source, we collected a dataset comprised of 60 GSL experiments with a teleoperated mobile robot [51]. The goal of the human operators was to identify and locate the gas source among several visually-identical candidate objects (see Figure 10). Results demonstrate that humans had over 75% success rate for search times between three to four minutes, supporting our hypothesis that semantic reasoning is indeed used by humans when locating the gas source with this configuration.

### 6.2. Semantic-Based Autonomous Gas Source Localization

The use of visual information when locating a gas source is not a novel approach, yet, it has been only superficially explored in the literature with very simple problem domains where the robot exploited prior knowledge about the source physical characteristics to reduce the locations to search [52]. Moreover, a formal way to define and exploit the relationships among gases and objects (i.e., their semantics) is still missing, aspect which could assist the GSL process in a more flexible way. In [53], as a partial result of the project, presented a novel GSL system that pursues both efficiency by exploiting the semantics between the detected gases and the objects in the environment, and coherence through the consideration of the uncertainty in the identification of gases and objects. To encode these semantic relationships (e.g., that heaters can release smoke), we rely on an ontology [39]. These factors make this approach particularly suitable for structured-indoor environments containing multiple objects likely to release gases where semantic relationships can be exploited.

Fusing the classification results (from both the detected gases and the recognized objects in the environment) together with the semantic information, a probabilistic Bayesian framework is proposed to assign to each detected object a probability of being the gas source. Finally, a path planning algorithm based on Markov Decision Processes (MDP) merges these probabilities with the navigation distances from the current robot location to the different objects (i.e., a cost value related to the time the robot would spend to reach the candidate object), to produce a plan that minimizes the search time. Both simulated (using computational fluid dynamic tools and GADEN gas dispersion simulator [54]) and real experiments demonstrate the feasibility of this novel approach by considerably reducing the search times and producing more coherent gas source searches.

## 7. Conclusions

In this paper, we have described and reviewed the goal and main contributions of the IRO project, focused on the improvement of the sensory and autonomous capability of mobile robots through olfaction.

We have first reviewed the concept of electronic nose, raising some specific issues when used on-board a mobile robot, and described a design of a modular e-nose suitable for mobile robotics applications. Then, having in mind the final goal of fusing different sensing modalities, we have focused on the intermediate tasks of visual object recognition and gas classification. Here, the project contribution consisted of different algorithms and experimental evaluations towards improving the recognition rates when these tasks are carried out with a mobile robot while navigating. Finally, we have introduced semantic reasoning to successfully fuse multiple sensing modalities when solving the challenging problem of gas source localization with a mobile robot. At this point, the project contributed with a novel architecture able to exploit the information provided by the vision and olfaction sensory sub-systems, as well as handling their respective uncertainties. For each detected object in the environment, a probability of being the gas source is estimated and afterward fed to a probabilistic framework that outputs the optimal path the robot should follow when inspecting the different objects in the environment, minimizing the search time.

## References

[1] Shepherd G.M. (2004). The human sense of smell: Are we better than we think?. PLoS Biol..

[2] Leite I., Martinho C., Paiva A. (2013). Social Robots for Long-Term Interaction: A Survey. Int. J. Soc. Robot..

[3] Truong X., Ngo T. (2018). “To Approach Humans?”: A Unified Framework for Approaching Pose Prediction and Socially Aware Robot Navigation. IEEE Trans. Cogn. Dev. Syst..

[4] Palacín J., Martínez D., Clotet E., Pallejà T., Burgués J., Fonollosa J., Pardo A., Marco S. (2019). Application of an array of Metal-Oxide Semiconductor gas sensors in an assistant personal robot for early gas leak detection. Sensors.

[5] Vincent T.A., Xing Y., Cole M., Gardner J.W. (2019). Investigation of the response of high-bandwidth MOX sensors to gas plumes for application on a mobile robot in hazardous environments. Sens. Actuators B Chem..

[6] Röck F., Barsan N., Weimar U. (2008). Electronic nose: Current status and future trends. Chem. Rev..

[7] Monroy J., Palomo E.J., Lopez-Rubio E., Gonzalez-Jimenez J. (2016). Continuous Chemical Classification in Uncontrolled Environments with Sliding Windows. Chemom. Intell. Lab. Syst..

[8] Gongora A., Monroy J., Gonzalez-Jimenez J. (2018). An Electronic Architecture for Multi-Purpose Artificial Noses. J. Sens..

[9] Sanchez-Garrido C., Monroy J., Gonzalez-Jimenez J. A Configurable Smart E-Nose for Spatio-Temporal Olfactory Analysis. Proceedings of the IEEE Sensors.

[10] Monroy J., Gonzalez-Jimenez J. (2018). Towards Odor-Sensitive Mobile Robots. Electronic Nose Technologies and Advances in Machine Olfaction.

[11] Ruiz-Sarmiento J.R., Galindo C., Gonzalez-Jimenez J. Probability and Common-Sense: Tandem Towards Robust Robotic Object Recognition in Ambient Assisted Living. Proceedings of the 10th International Conference on Ubiquitous Computing and Ambient Intelligence.

[12] Galindo C., Saffiotti A. (2013). Inferring robot goals from violations of semantic knowledge. Robot. Auton. Syst..

[13] Gutierrez-Osuna R., Nagle H.T. (1999). A method for evaluating data-preprocessing techniques for odour classification with an array of gas sensors. IEEE Trans. Syst. Man Cybern. Part B.

[14] Gunter A.T., Koren V., Chikkadi K., Righettoni M., Pratsinis S.E. (2016). E-nose sensing of low-ppb formaldehyde in gas mixtures at high relative humidity for breath screening of lung cancer. Acs Sens..

[15] Monroy J., Lilienthal A., Blanco J.L., Gonzalez-Jimenez J., Trincavelli M. (2013). Probabilistic Gas Quantification with MOX Sensors in Open Sampling Systems—A Gaussian Process Approach. Sens. Actuators B Chem..

[16] Kim H.J., Lee J.H. (2014). Highly sensitive and selective gas sensors using p-type oxide semiconductors: Overview. Sens. Actuators B Chem..

[17] Ponzoni A., Comini E., Sberveglieri G., Zhou J., Deng S.Z., Xu N.S., Ding Y., Wang Z.L. (2006). Ultrasensitive and highly selective gas sensors using three-dimensional tungsten oxide nanowire networks. Appl. Phys. Lett..

[18] Cui S., Wang J., Yang L., Wu J., Wang X. (2015). Qualitative and quantitative analysis on aroma characteristics of ginseng at different ages using E-nose and GC–MS combined with chemometrics. J. Pharm. Biomed. Anal..

[19] Monroy J., Gonzalez-Jimenez J., Blanco J.L. (2012). Overcoming the slow recovery of MOX gas sensors through a system modeling approach. Sensors.

[20] Gonzalez-Jimenez J., Monroy J., Blanco J.L. (2011). The Multi-Chamber Electronic Nose–An Improved Olfaction Sensor for Mobile Robotics. Sensors.

[21] Coradeschi S., Cesta A., Cortellessa G., Coraci L., Galindo C., Gonzalez-Jimenez J., Karlsson L., Forsberg A., Frennert S., Furfari F. (2014). GiraffPlus: A System for Monitoring Activities and Physiological Parameters and Promoting Social Interaction for Elderly. Human-Computer Systems Interaction: Backgrounds and Applications 3.

[22] Luperto M., Monroy J., Ruiz-Sarmiento J.R., Moreno F.A., Basilico N., Gonzalez-Jimenez J., Borghese N.A. Towards Long-Term Deployment of a Mobile Robot for at-Home Ambient Assisted Living of the Elderly. Proceedings of the European Conference on Mobile Robots.

[23] Trincavelli M., Coradeschi S., Loutfi A. (2009). Odour classification system for continuous monitoring applications. Sens. Actuators B Chem..

[24] Onkal-Engin G., Demir I., Engin S.N. (2005). Determination of the relationship between sewage odour and BOD by neural networks. Environ. Model. Softw..

[25] Monroy J., Gonzalez-Jimenez J., Sanchez-Garrido C. Monitoring Household Garbage Odors in Urban Areas Through Distribution Maps. Proceedings of the IEEE Sensors.

[26] Hasenfratz D., Saukh O., Walser C., Hueglin C., Fierz M., Arn T., Beutel J., Thiele L. (2015). Deriving high-resolution urban air pollution maps using mobile sensor nodes. Pervasive Mob. Comput..

[27] Guo D., Zhang D., Li N., Zhang L., Yang J. (2010). A novel breath analysis system based on electronic olfaction. IEEE Trans. Biomed. Eng..

[28] Gibb K.A., Yee A.S., Johnston C.C., Martin S.D., Nowak R.M. (1984). Accuracy and usefulness of a breath alcohol analyzer. Ann. Emerg. Med..

[29] Hlastala M.P. (1998). The alcohol breath test—A review. J. Appl. Physiol..

[30] Marques L., Nunes U., de Almeida A.T. (2002). Olfaction-based mobile robot navigation. Thin Solid Film..

[31] Vergara A., Fonollosa J., Mahiques J., Trincavelli M., Rulkov N., Huerta R. (2013). On the performance of gas sensor arrays in open sampling systems using Inhibitory Support Vector Machines. Sens. Actuators B Chem..

[32] Trincavelli M. (2011). Gas discrimination for mobile robots. KI-Künst. Intell..

[33] Fu T.C. (2011). A review on time series data mining. Eng. Appl. Artif. Intell..

[34] Schleif F.M., Hammer B., Monroy J., Gonzalez-Jimenez J., Blanco J.L., Biehl M., Petkov N. (2016). Odor recognition in robotics applications by discriminative time-series modeling. Pattern Anal. Appl..

[35] Monroy J., Gonzalez-Jimenez J. (2017). Gas Classification in Motion: An Experimental Analysis. Sens. Actuators B. Chem..

[36] Galleguillos C., Belongie S. (2010). Context Based Object Categorization: A Critical Survey. Comput. Vis. Image Underst..

[37] Oliva A., Torralba A. (2007). The role of context in object recognition. Trends Cogn. Sci..

[38] Koller D., Friedman N. (2009). Probabilistic Graphical Models: Principles and Techniques-Adaptive Computation and Machine Learning.

[39] Uschold M., Gruninger M. (1996). Ontologies: Principles, methods and applications. Knowl. Eng. Rev..

[40] Ruiz-Sarmiento J.R., Galindo C., Gonzalez-Jimenez J. (2017). A survey on learning approaches for Undirected Graphical Models. Application to scene object recognition. Int. J. Approx. Reason..

[41] Murphy K.P., Weiss Y., Jordan M.I. Loopy Belief Propagation for Approximate Inference: An Empirical Study. Proceedings of the Fifteenth Conference on Uncertainty in Artificial Intelligence.

[42] Ruiz-Sarmiento J.R., Galindo C., Gonzalez-Jimenez J. (2017). Robot@Home, a robotic dataset for semantic mapping of home environments. Int. J. Robot. Res..

[43] Ruiz-Sarmiento J.R., Galindo C., Gonzalez-Jimenez J. (2017). Building Multiversal Semantic Maps for Mobile Robot Operation. Knowl. Based Syst..

[44] Galindo C., Saffiotti A., Coradeschi S., Buschka P., Fernandez-Madrigal J.A., Gonzalez J. Multi-hierarchical semantic maps for mobile robotics. Proceedings of the 2005 IEEE/RSJ International Conference on Intelligent Robots and Systems.

[45] Kostavelis I., Gasteratos A. (2015). Semantic mapping for mobile robotics tasks: A survey. Robot. Auton. Syst..

[46] Kam M., Zhu X., Kalata P. (1997). Sensor fusion for mobile robot navigation. Proc. IEEE.

[47] Kowadlo G., Russell R.A. (2008). Robot odor localization: A taxonomy and survey. Int. J. Robot. Res..

[48] Monroy J., Melendez-Fernandez F., Gongora A., Gonzalez-Jimenez J. Integrating Olfaction in a Robotic Telepresence Loop. Proceedings of the 2017 26th IEEE International Symposium on Robot and Human Interactive Communication (RO-MAN).

[49] Monroy J., Ruiz-Sarmiento J.R., Moreno F.A., Galindo C., Gonzalez-Jimenez J. Towards a Semantic Gas Source Localization under Uncertainty. Proceedings of the International Conference on Information Processing and Management of Uncertainty in Knowledge-Based Systems.

[50] Gongora A., Monroy J., Gonzalez-Jimenez J. A Robotic Experiment Toward Understanding Human Gas-Source Localization Strategies. Proceedings of the 2017 ISOCS/IEEE International Symposium on Olfaction and Electronic Nose (ISOEN).

[51] Gongora A., Gonzalez-Jimenez J. (2019). Olfactory telerobotics. A feasible solution for teleoperated localization of gas sources?. Robot. Auton. Syst..

[52] Ishida H., Tanaka H., Taniguchi H., Moriizumi T. (2006). Mobile robot navigation using vision and olfaction to search for a gas/odor source. Auton. Robot..

[53] Monroy J., Ruiz-Sarmiento J.R., Moreno F.A., Melendez-Fernandez F., Galindo C., Gonzalez-Jimenez J. (2018). A Semantic-Based Gas Source Localization with a Mobile Robot Combining Vision and Chemical Sensing. Sensors.

[54] Monroy J., Hernandez-Bennetts V., Fan H., Lilienthal A., Gonzalez-Jimenez J. (2017). GADEN: A 3D Gas Dispersion Simulator for Mobile Robot Olfaction in Realistic Environments. Sensors.

